# Assessment of Uterocervical Angle Width as a Predictive Factor of Preterm Birth: A Systematic Review of the Literature

**DOI:** 10.1155/2018/1837478

**Published:** 2018-12-26

**Authors:** George Daskalakis, Marianna Theodora, Panagiotis Antsaklis, Michael Sindos, Themistoklis Grigoriadis, Aris Antsaklis, Nikolaos Papantoniou, Dimitrios Loutradis, Vasilios Pergialiotis

**Affiliations:** ^1^First Department of Obstetrics and Gynecology, Alexandra Hospital, National and Kapodistrian University of Athens, Greece; ^2^Third Department of Obstetrics and Gynecology, Attikon University Hospital, National and Kapodistrian University of Athens, Greece

## Abstract

**Background:**

Uterocervical angle (UCA) has been recently proposed as a potential marker that could accurately predict preterm birth (PTB). The purpose of the present systematic review is to accumulate current evidence and provide directions for future research.

**Materials and Methods:**

We used the Medline (1966–2018), Scopus (2004–2018), Clinicaltrials.gov (2008–2018), EMBASE (1980-2018), Cochrane Central Register of Controlled Trials CENTRAL (1999-2018), and Google Scholar (2004-2018) databases in our search.

**Results:**

Eleven studies were finally included in the present systematic review that evaluated data from 3,018 women. The significant heterogeneity in terms of outcome reporting and outcome reporting measures (use of optimal cut-off values) precluded meta-analysis. However, existing data support that second trimester UCA measurement might be used as a predictive factor of PTB <34 weeks, as at least two studies in unselected singleton pregnancies and two studies in pregnancies with an ultrasonographically shortened cervix seem to support this hypothesis. The most commonly reported cut-off values were 105° and 95°.

**Conclusions:**

UCA measurement during the second trimester of pregnancy may be a useful method of determining women at risk of delivering preterm. However, more studies are needed to assess the reproducibility of these findings and reach conclusive evidence.

## 1. Introduction

Preterm birth (PTB) is a leading cause of perinatal morbidity and mortality and is estimated to complicate approximately 10-12% of pregnancies [1]. To date, the optimal strategy of pregnancies at risk of preterm birth remains unclear. Progesterone, cervical cerclage, and the Arabin pessary have been used as potential management strategies in women with singleton pregnancies with a short cervix and history of previous spontaneous preterm birth [2]. In a recent network meta-analysis Jarde et al. observed that progesterone seems to be the best intervention; however, the significant heterogeneity of included studies precluded safe interpretation of their findings [3]. Screening of pregnancies remains also problematic as the majority of current strategies is far from an optimal diagnostic accuracy. Fetal fibronectin has been suggested as a potential biomarker for the prevention of preterm birth; however, its sensitivity is relatively low (34%) [4]. Current data also suggest that cervical length (CL) measurement may help identify these women as it may accurately predict pregnancies at risk of preterm birth [5, 6]. In this line, current guidelines suggest that women with a history of spontaneous preterm delivery or second trimester loss, as well as those with a short cervix (<25 mm) in a transvaginal ultrasound scan between 16 and 24 weeks of gestation should be offered treatment with cerclage or progesterone [7].

Uterocervical angle (UCA) represents a novel ultrasonographic marker that is defined as the triangular segment measured between the lower uterine segment and the cervical canal. It is measured using a line that starts from the internal cervical os (that is extended along the cervical canal) and a second line that tracks the internal segment of the anterior uterine wall. During the last years several studies investigated the potential impact of UCA for the prediction of preterm birth. The rationale behind the hypothesis of this association is based on the potential mechanical properties of this angle, which seems to act as a preventive barrier when it is acute. The first article that supported this assumption was written by Cannie et al. who supported that the efficacy of the Arabin pessary in preventing preterm birth was significantly influenced by the change in the UCA pre- and postpessary insertion [8]. Keepanasseril et al. also suggested that UCA may be a mechanical barrier that might influence the progress of labour [9]. These authors supported at 2007 that a posterior cervical angle of at least 100° is accompanied by a specificity and specificity of 65% and 72%, respectively, for the prediction of successful induction of labour in nulliparous women. Assuming that this angle might be also predictive in determining women at risk for preterm birth a significant number of articles were published. The purpose of the present systematic review is to accumulate and present current evidence in this field and to provide recommendations for future research.

## 2. Materials and Methods

The present systematic review was designed according to the Preferred Reporting Items for Systematic Reviews and Meta-Analyses (PRISMA) guidelines [10].

### 2.1. Information Sources and Search Methods

**Figure 1:**
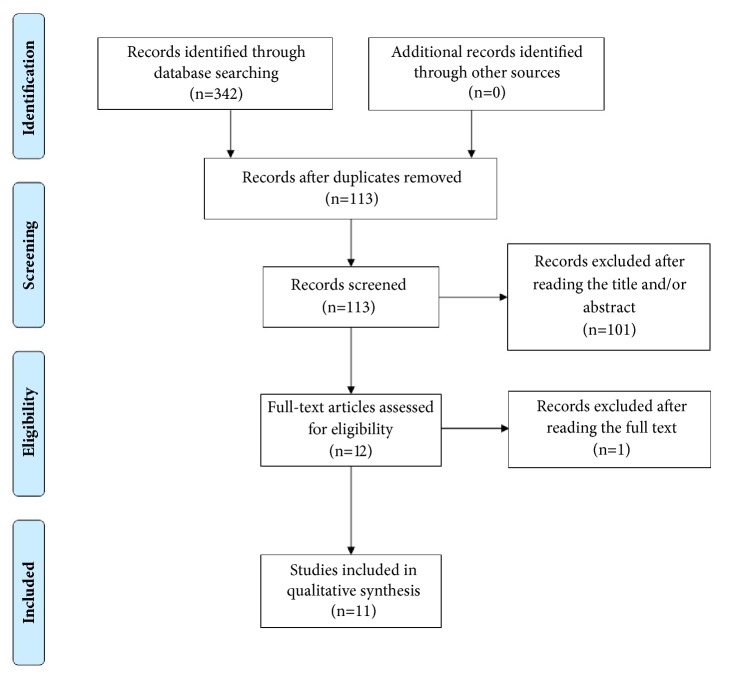
Search plot diagram.

We used the Medline (1966–2018), Scopus (2004–2018), Clinicaltrials.gov (2008–2018), EMBASE (1980-2018), Cochrane Central Register of Controlled Trials CENTRAL (1999-2018), and Google Scholar (2004-2018) databases in our primary search along with the reference lists of electronically retrieved full-text papers. The date of our last search was set at 28 February 2018. Our search strategy included the text words “*angle, preterm, cervix, cervical*” and is schematically presented in the PRISMA flow diagram (Figure 1).

The studies were selected in three consecutive stages. Following deduplication, the titles and abstracts of all electronic articles were screened by two authors (V. P. and G. D.) to assess their eligibility. The decision for inclusion of studies in the present systematic review was taken after retrieving and reviewing the full text of articles that were held potentially eligible. Potential discrepancies in this latter stage were resolved by the consensus of all authors.

### 2.2. Quality and Risk of Bias Assessment

The risk of bias and methodological quality of the included studies was explored using the Newcastle-Ottawa Scale (NOS), which evaluates the selection of the study groups, the comparability of the groups, and the ascertainment of the exposure or outcome of interest [11].

### 2.3. Study Selection

#### 2.3.1. Types of Studies and Patients

The eligibility criteria for the inclusion of studies were predetermined. No language restrictions were applied. All observational studies as well as randomized trials that assessed the differences and, whenever present, the predictive accuracy of UCA in preterm birth were held eligible for inclusion (irrespective of the existence of other variables from the patients' history including preterm premature rupture of membranes (PPROM), previous preterm births, parity, gravidity, and singleton/multiple gestation). Conference abstracts were also included. Case reports as well as experimental animal studies and reviews were not included in the qualitative analysis.

#### 2.3.2. Outcome Measures

The mean difference in uterocervical angle among pregnancies delivered at term and preterm (<37 weeks of gestation) was predefined as primary outcome measure. The sensitivity and specificity of UCA in detecting pregnancies at risk of delivering prior to the 37th, 34th, 32nd, and 28th week of gestation were also defined as primary outcome measures.

Secondary outcome measures were defined following completion of data extraction and included differences in gestational latency period following PPROM and latency period following cerclage placement.

## 3. Results

Eleven studies were finally included in the present systematic review that evaluated data from 3,018 women (Table 1) [12–22]. Among them, 5 studies evaluated pregnancy outcomes in unselected singleton pregnancies [14, 16, 17, 21, 22], two studies reported outcomes in women with a shortened cervix that were offered cerclage [6, 21], one study evaluated changes in UCA in women with a shortened cervix that were followed up with at least two measurements of cervical length during the second trimester of pregnancy [15], two studies enrolled unselected twin pregnancies [13, 18], and one study evaluated the impact of UCA in the latency period of pregnancies complicated by PPROM [20]. The results of the Newcastle-Ottawa Scale are presented in Table 2. In general, the quality of most studies was evaluated as fair–high; however, their comparability was evaluated as inappropriate as none of the included cohorts presented adjusted analysis according to the cervical angle (a factor that has already been described as predisposing for preterm birth). Only one study evaluated the role of confounders other than cervical length (including maternal age, nulliparity, race, and obesity) on uterocervical angle [22]. The single case control study that was included in the present systematic review scored three stars for patient selection, no stars for comparability, and three stars for exposure [21].

### 3.1. Outcomes in Unselected Singleton Gestations

Sur et al. found significant differences in mean UCA between pregnancies that delivered preterm (<37 weeks) and those that delivered at term, during both the first (114.2° versus 93.0°) and second trimester of pregnancy (127.66° versus 103.65°) [14]. In line with this observation was the study of Faras-Llobet et al. that suggested that the angle during the second trimester screening was significantly wider in women that delivered at <34 weeks compared to those that delivered at term (105.16° versus 94.53°) [5]. In another study, however, the same group of researchers reported there is a minimal difference among women that delivered at term compared to those that delivered preterm (<37 weeks) (103.6° versus 101.7°), implying that at least second trimester measurement may not be as predictive as we would like to think so [13]. Martinez et al. also confirmed that pregnant women with a wide UCA are prone to deliver preterm (<34 weeks) compared to women that delivered at term (106.1° versus 99.5°) [21]. They also mentioned that UCA was independent of the CL measurement and could thus be used in predictive models combined with CL expressed as multiples of the median (MoM), maternal characteristics, and history (maternal age and history of previous PTB). Finally, Dziadosz et al. observed that second trimester UCA measurement could detect the possibility of preterm birth <37 weeks with a sensitivity of 80% when the angle was ≥95° and <34 weeks with a sensitivity of 81% when the angle was ≥105° [22]. The same authors also reported that when they performed stepwise linear regression analysis they observed that UCA was dependent on maternal age, obesity at conception, nulliparity, and race.

### 3.2. Outcomes in Pregnancies with an Ultrasonographically Shortened Cervix

Swanson et al. investigated the accuracy of UCA in predicting gestational latency in women with physical examination indicated cerclage [12]. They used predetermined cut-off values of UCA at 95° and 105° to stratify patients and used ultrasound images that were obtained prior to cerclage placement. No differences were noted and the authors concluded that UCA cannot predict gestational latency in women undergoing physical examination indicated cerclage. On the other hand, Knight et al. suggested that UCA angle prior to delivery was predictive of cerclage failure [19]. However, this was not the case with UCA angle estimation prior to cerclage placement or shortly after cerclage placement. The authors used optimal cut-offs to estimate the potentially predictive accuracy of the method and reported that a cut-off of 108° was able to detect delivery prior to 34 weeks with a sensitivity of 97% and specificity of 65%. Concerning delivery prior to 28 weeks the optimal performance of the ROC analysis was observed at 112° with a sensitivity of 100% and a specificity of 62%. Finally, Lynch et al. evaluated women that were sequentially screened during the second trimester of pregnancy and observed that the difference in UCA among these measurements was not able to predict preterm birth [15]. However, they did mention that a final UCA of ≥105° prior to 25 weeks of gestation was associated with an increased risk of preterm birth <34 weeks (24.2% versus 6.8%,* p=.01*).

### 3.3. Outcomes in Twin Pregnancies

As previously mentioned only two studies reported outcomes in unselected twin pregnancies that were ultrasonographically evaluated during the second trimester. Lynch et al. used predetermined cut-off values of 95° and 105° and observed that they were both associated with an increased risk of PTB <37 weeks ((55.9% versus 31.6%,* p=.05* and 58.3% versus 35.3%,* p=.02*, respectively) [13]. The authors also compared performance metrics of UCA with CL and observed that UCA was accompanied by significant sensitivity (exceeding 80%) but low specificity (less than 35%), whereas CL was more specific (98.5%) but less sensitive (12.5%). Knight et al. also evaluated second trimester UCA and observed that the use of optimal cut-offs resulted in enhanced predictive accuracy compared to CL measurement (<20mm) for PTB <34 and <28 weeks [18]. Specifically, the cut-off of 110° was accompanied by 80% sensitivity and 82% specificity for the prediction of PTB <34 weeks.

### 3.4. Outcomes from Other Published Studies

A study was published at 2017 by Kathir et al. that investigated whether CL and UCA were associated with gestational latency in women with PPROM [20]. The authors reported that whereas CL did not influence the latency period in these cases UCA exerted a mild effect that requires further investigation in the future (Hazard ratio 1.03, 95% CI 1.01 – 1.06,* p=.003*).

## 4. Discussion

Existing data support that second trimester UCA measurement might be used as a predictive factor of PTB <34 weeks, given that at least two studies in unselected singleton pregnancies, and two studies in pregnancies with an ultrasonographically shortened cervix seem to support this hypothesis. However, to date most of the available evidence is based in optimal cut-off values; hence the accumulation of data in a meta-analytic approach remains out of the question.

The actual factors that influence this angle, however, remain, to date, unknown. Preconceptional UCA is directly related to uterine version and flexion and this factor should be evaluated in large future cohorts. Constitutional changes in the physiology of the cervix during pregnancy may also affect the flexibility of the cervix and significantly modify UCA. At 2012 Heller et al. evaluated uterine cervices from 22 cases of obstetric hysterectomy and observed that the mean percent of collagen was significantly higher in cervices on nonpregnant uteri compared to pregnant uteri (73.5±3.5% versus 21.5±2.2%) [23]. This study indicates the presence of significant differences in the physiology of the cervix during pregnancy. Recently, Sundtoft et al. also suggested that women with cervical insufficiency have lower collagen concentrations (63.5 ± 5.1%) compared with controls (68.2 ± 5.4%)* p<.001* [24]. None of the existing studies evaluates directly the impact of the cervical microenvironment on the UCA. However, a recent indirect comparison between collagen fiber orientation and dispersion in the upper cervix of pregnant and nonpregnant women suggested that collagen fiber dispersion and direction may influence cervical remodeling during pregnancy [25].

The main strength of the present systematic review is the accumulation for the first time in the international literature of evidence related to the diagnostic accuracy of UCA for the prediction of preterm birth. The majority of included studies scored high for patient selection and outcome reporting; hence current data can be considered for the conduct of future research in this field. On the other hand, the wide heterogeneity in terms of the selected population, outcome reporting (UCA cut-off value), and outcome of interest (gestational week that was used as a cut-off value of preterm birth) among the included studies rendered impossible the conduct of a meta-analysis of diagnostic accuracy (Table 1).

Taking this information into consideration and despite the potential pathophysiological background that was already mentioned, existing evidence, although promising, does not suffice to introduce UCA in current clinical practice as a predictive factor that may be used for decision-making regarding management of women at risk of delivering preterm. This is why, future studies are needed to evaluate the diagnostic accuracy of this index, and these should specifically consider the use of cut-off values and outcomes of interest (preterm birth rates based on specific gestational weeks) that were presented in the present systematic review. Moreover, they should adjust their findings according to the CL as it remains unknown whether an overlap between CL and UCA exists that might influence the detection rate of the latter index.

## Figures and Tables

**Table 1:** Methodological characteristics and patient selection in included studies (PEIC, physical examination indicated cerclage; PPROM, preterm premature rupture of the membranes).

***Study characteristics***				
***Year; author***	***Study design***	***Patient n***	***Inclusion criteria***	***Outcomes of interest***
2018; Swanson	Retrospective cohort	60	Ultrasound examination no more than 3 weeks prior to PEIC (length <2cm and dilatation)	Gestational latency period
2018; Lynch	Retrospective cohort	137	Unselected twin pregnancies that had an ultrasound scan between 14 and 25 weeks.	Spontaneous preterm birth (<37 weeks)
2017; Sur	Prospective cohort	100	Women with singleton uncomplicated pregnancy scanned during the 1st and 2nd trimester	Spontaneous preterm birth (<37 weeks)
2017; Lynch	Retrospective cohort	176	Women with singleton pregnancy and CL<25mm between 14 and 25 weeks. Women with only 1 measurement of CL were excluded	Rates of spontaneous preterm birth (<37 weeks) in women with a short cervix
2017; Farras Llobet poster	Prospective study	499	Unselected singleton pregnancies that had an ultrasound scan between 18 and 24 weeks.	Spontaneous preterm birth (<37 weeks)
2017; Farras Llobet	Prospective case control	275	Unselected singleton pregnancies that had an ultrasound scan between 18 and 24 weeks.	Spontaneous preterm birth (<34 weeks)
2017; Knight (2)	Retrospective cohort	259	Twin pregnancies that had an ultrasound scan between 16 and 23 weeks.	Spontaneous preterm birth (<32 weeks and <28 weeks)
2017; Knight	Retrospective cohort	142	Women with PEIC that had ultrasound examination 1 week after cerclage placement	Preterm birth (<34 weeks, <28 weeks)
2017; Kathir	Prospective cohort	80	Women with singleton pregnancy between 28 and 34 weeks, PPROM, not in labour	Pregnancy latency period
2016; Martinez	Retrospective nested case control	318	Unselected singleton pregnancies that had an ultrasound scan between 14 and 24 weeks.	Spontaneous preterm birth (<34weeks)
2016; Dziadosz	Retrospective cohort	972	Women with singleton pregnancy that had an ultrasound scan between 16 and 24 weeks	Spontaneous preterm birth (<37 weeks and <34 weeks)

**Table 2:** Newcastle-Ottawa scale score of included studies.

**Newcastle-Ottawa Assessment Scale**									
**Date; Author**	**Selection**				**Comparability**	**Outcome**			**Total**
	Representativeness of the exposed cohort	Selection of the non- exposed cohort	Ascertainment of exposure	Outcome of interest not present at start of study		Assessment of outcome	Adequacy of duration of follow up	Adequacy of completeness of follow up	
2018; Swanson	-	★	★	★	-	★	★	★	6
2018; Lynch	★	★	?	?	-	?	★	★	4
2017; Sur	★	★	★	★	-	★	★	★	7
2017; Lynch	★	★	★	★	-	★	★	★	7
2017; Farras Llobet poster	★	★	★	★	-	?	★	★	6
2017; Farras Llobet	-	★	★	★	-	★	★	★	6
2017; Knight (2)	★	★	★	★	-	★	★	★	7
2017; Knight	★	★	★	★	-	★	★	★	7
2017; Kathir	★	★	★	★	-	★	★	★	7
2016; Dziadosz	★	★	★	★	★	★	★	★	8
